# Quantifying the benefits of using decision models with response time and accuracy data

**DOI:** 10.3758/s13428-020-01372-w

**Published:** 2020-03-30

**Authors:** Tom Stafford, Angelo Pirrone, Mike Croucher, Anna Krystalli

**Affiliations:** 1 Department of Psychology, University of Sheffield, 1 Vicar Lane, Sheffield, S1 2LT UK; 2 Centre for Philosophy of Natural and Social Science, London School of Economics and Political Science, London, UK; 3 Numerical Algorithms Group Ltd, Oxford, UK; 4 Research Software Engineering, University of Sheffield, Sheffield, UK

**Keywords:** Speed-accuracy trade-off, Drift-diffusion model, Statistical power, Response time, Accuracy

## Abstract

Response time and accuracy are fundamental measures of behavioral science, but discerning participants’ underlying abilities can be masked by speed–accuracy trade-offs (SATOs). SATOs are often inadequately addressed in experiment analyses which focus on a single variable or which involve a suboptimal analytic correction. Models of decision-making, such as the drift diffusion model (DDM), provide a principled account of the decision-making process, allowing the recovery of SATO-unconfounded decision parameters from observed behavioral variables. For plausible parameters of a typical between-groups experiment, we simulate experimental data, for both real and null group differences in participants’ ability to discriminate stimuli (represented by differences in the drift rate parameter of the DDM used to generate the simulated data), for both systematic and null SATOs. We then use the DDM to fit the generated data. This allows the direct comparison of the specificity and sensitivity for testing of group differences of different measures (accuracy, reaction time, and the drift rate from the model fitting). Our purpose here is not to make a theoretical innovation in decision modeling, but to use established decision models to demonstrate and quantify the benefits of decision modeling for experimentalists. We show, in terms of reduction of required sample size, how decision modeling can allow dramatically more efficient data collection for set statistical power; we confirm and depict the non-linear speed–accuracy relation; and we show how accuracy can be a more sensitive measure than response time given decision parameters which reasonably reflect a typical experiment.

## Introduction

### Speed–accuracy trade-offs

Speed and accuracy of responding are fundamental measures of performance, collected by behavioral scientists across diverse domains in an attempt to track participants’ underlying capacities. As well as being affected by the capacity of participants to respond quickly and accurately, the two measures are also related by participants’ strategic choices of a speed–accuracy trade-off (SATO; for reviews see Heitz, 2014; Wickelgren, 1977).

The SATO confounds measurement of participant capacity—which means that we cannot directly read either speed or accuracy as an index of participant ability. The SATO is inherent to decision-making—it arises whenever we wish to respond as fast and as accurately as possible based on uncertain incoming information. More accurate responses require more information, which takes longer to accumulate; faster responses forgo collecting additional information at the cost of higher error rates. Importantly, because the SATO is unavoidable, it is also necessary that all decision-making processes are positioned with respect to the trade-off. This does not need to be done deliberately or explicitly, but any decision process can be characterized as adopting some trade-off between speed and accuracy. For the tasks studied by psychologists, it is important to recognize that there will be individual differences, as well as task and group-related differences, in how participants position themselves on the SATO.

Outside of research focused on SATOs explicitly, different practices have been adopted to account for SATOs or potential SATOs in behavioral data. One approach is to ignore either speed or accuracy. For example, ignoring speed of response is common in psychophysics, whereas some domains of cognitive psychology where high accuracy is assumed, focus only on response times (e.g., Stafford, Ingram, & Gurney, 2011),^1^ albeit sometimes after a cursory check that standard null-hypothesis tests do not reveal significant differences in error rates. Another approach is to combine speed and accuracy. For example, in the domain of visual search it is common to calculate ‘efficiency’ scores by dividing search time by search accuracy as a proportion (e.g., Yates & Stafford, 2018, June). Despite being widespread, there is evidence that this practice is unlikely to add clarity to analysis (Bruyer & Brysbaert, 2011). We also note that the researchers who initially formulated the efficiency score explicitly counseled *against* using it in the case of SATOs (Townsend & Ashby, 1983).

The efficiency score shares the property with other recent suggestions for accounting for SATOs (Davidson & Martin, 2013; Seli, Jonker, Cheyne, & Smilek, 2013) that it assumes a linear relation between response time and accuracy. While such approaches may be better than focusing on a single behavioral variable, the assumption of linearity is at odds with work which has explicitly characterized the SATO (Fitts, 1966; Heitz, 2014; Wickelgren, 1977) and has shown a distinctly curvilinear relation between response time and accuracy. As such, although linear correction methods may work for some portions of the SATO curve, they are likely to be misleading, or at least fail to add clarity, where accuracy and/or speed approaches upper or lower limits of those variables. Recently, Liesefeld and Janczyk (2019) showed that several current methods for combing speed and accuracy to correct for SATOs are in fact sensitive to the very SATOs they are designed to account for. These authors advocate the balanced integration score (BIS; Liesefeld, Fu, & Zimmer, 2015) as an alternative, but it seems likely that the combination of speed and accuracy remains an estimation problem of some delicacy, especially in the presence of SATOs.

### Context

The unprincipled combination of speed and accuracy measures becomes an urgent issue when considered in the context of widespread questions surrounding the reliability of the literature in psychology. Established results fail to replicate, or replicate with substantially reduced effect sizes (Open Science Collaboration, 2015; Pashler & Wagenmakers, 2012).

Low statistical power has been a persistent problem across many areas of psychology and cognitive neuroscience (Button et al.,, 2013; Lovakov & Agadullina, 2017, November; Maxwell, 2004; Sedlmeier & Gigerenzer, 1989; Stanley, Carter, & Doucouliagos, 2017; Szucs & Ioannidis, 2017), including, but not limited to, research areas which are bound by costly methods or hard-to-reach populations (Bezeau & Graves, 2001; Cohen, 1962; Geuter, Qi, Welsh, Wager, & Lindquist, 2018). This, combined with factors such as analytic flexibility (Silberzahn et al.,, 2017; Simmons, Nelson, & Simonsohn, 2011)—which can only be increased by the lack of a single standard method for accounting for SATOs—has led to a widespread loss of faith in many published results (Ioannidis, 2005).

Statistical power is defined with respect to the variability and availability of data, as well as the analysis proposed. For a set experimental design, an obvious candidate for increasing statistical power is to increase sample size, but this is not always easy. Each additional participant costs additional time, money and resources. This is especially true in the case of expensive methods, such as fMRI, or special populations which may be hard to recruit. More sensitive measures also increase statistical power: lower measurement error will tend to reduce variability so that the same mean differences produce larger observed effect sizes.

A motivation for the present work is to demonstrate the practical utility, in terms of increased statistical power, of combining speed and accuracy information in a principled manner using decision models. Such an innovation has the appeal of making the most of data which is normally collected, even if not analyzed, while not requiring more participants (which is costly), or more trials per participant (which also has costs in terms of participant fatigue, which may be especially high for some populations, e.g., children).

### Decision modeling

Models of the decision-making process provide the foundation for the principled combination of speed and accuracy data, and thus afford experimenters access to considerable statistical power gains.

Many models exist in which decision-making is represented by the accumulation of sensory evidence over time. When the accumulated evidence surpasses some threshold (also called a boundary) then a decision is triggered. The accuracy of the decision depends on which accumulator crosses which boundary; the speed is given by time this takes, and thus such models can be used to fit speed and accuracy data within the same framework.

A prominent instance of such accumulator models is the so called drift-diffusion model developed by Roger Ratcliff (DDM, Ratcliff, 1978; Ratcliff & Rouder, 1998). In these models, the rate at which evidence is accumulated is represented by the drift rate parameter, which can be thought of as co-determined by the sensitivity of perceiver and the strength of the stimulus. After a long and successful period of development and application on purely behavioral data, the DDM model was at the center of an important theoretical confluence. Neurophysiologists found evidence for accumulation like processes in neurons critical to sensory decision-making (Gold & Shadlen, 2001; Smith & Ratcliff, 2004), while theoreticians recognized that accumulator models could be related to statistical methods of uncertain information integration. Under certain parameterizations, many different decision models, all in the family of accumulator models, can be shown to be equivalent to the DDM, and thus in turn equivalent to a statistical method which is optimal for making the fastest decision with a given error rate, or the most accurate decision within a fixed time (Bogacz, Brown, Moehlis, Holmes, & Cohen, 2006; Gold & Shadlen, 2002).

**Fig. 1:**
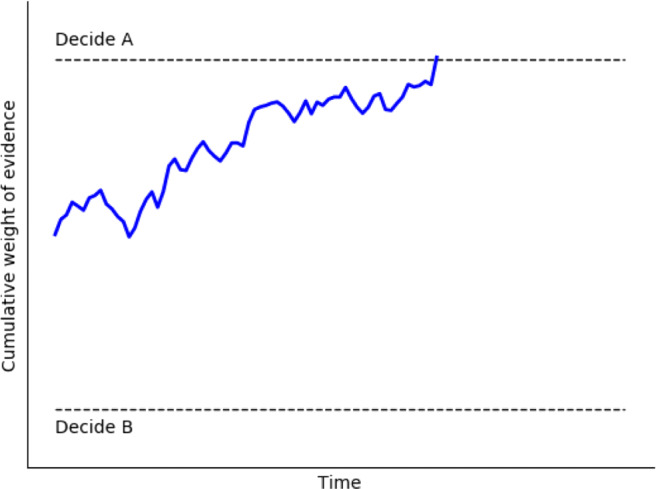
Decision-making by evidence accumulation. Here, a single possible accumulation process is shown (in *blue*). The rate of accumulation is determined by incoming sensory evidence as well as Gaussian noise. The response is determined when the accumulator value on the *y*-axis crosses the upper or lower threshold (*dashed lines*; in this case, Decision A is triggered). The response time is determined by the distance the accumulator travels on the *x*-axis (time)

While debate continues around the exact specification of the decision model which best reflects human decision-making, there is a consensus that the DDM captures many essential features of decision processing (but see Pirrone, Azab, Hayden, Stafford, & Marshall, 2018; Pirrone, Stafford, & Marshall, 2014; Teodorescu, Moran, & Usher, 2016). As you would expect, the DDM has also shown considerable success modeling decision data across many different domains (Ratcliff, Smith, & McKoon, 2015; Ratcliff, Smith, Brown, & McKoon, 2016), and in particular at separating out response thresholds from stimulus perception (Ratcliff & McKoon, 2008), and in estimating these reliably (Lerche & Voss, 2017). In the sense that the DDM implements a statistically optimal algorithm for accumulation for uncertain information, we would expect our neural machinery to implement the same algorithm in the absence of other constraints (Pirrone et al., 2014). The basic mechanism of the DDM is that of a single accumulator, similar to that shown in Fig. 1, with the following key parameters: *v*, the drift rate which reflects the rate of evidence accumulation; *a*, the boundary separation, which defines the threshold which must be crossed to trigger a decision and so reflect response conservativeness; *z*, the starting point of accumulation (either equidistant between the two decision thresholds, or closer to one rather than the other), which biases the response based on pre-stimulus expectations and *T*_*er*_, non-decision time, a fixed delay which does not vary with stimulus information. Additional parameters define noise factors which set factors such as the trial-to-trial variability in drift rate.

For our purposes, the value of these decision models is that they provide a principled reconciliation of speed and accuracy data. Within this framework, these observed behavioral measures reflect the hidden parameters of the decision model, most important of which are the drift rate (reflecting the rate of evidence accumulation) and the decision boundary separation (reflecting the conservativeness of the participant’s decision criterion; higher boundaries produce slower but more accurate responses).

By fitting the DDM to our data, we can deconfound the observed behavioral variables—speed and accuracy—and recover the putative generating parameters of the decision—drift and boundary separation. In principle, this allows a more sensitive measure of participant capability (reflected in the drift parameter). Drift is a more sensitive measure because (a) it is estimated using both speed *and* accuracy, (b) this estimation takes account of both mean response time and the distribution of response times for correct and error responses, and because (c) the estimation of the drift parameter is isolated from the effect of different participant’s SATOs (which are reflected in the boundary parameter).

### Prior work

Previous authors have established the principled benefits of this approach (Ratcliff & McKoon, 2008). Within a psychophysics framework, Stone (2014) extended Palmer, Huk, and Shadlen (2005)’s decision model to show that response time and accuracy contain different, but possibly overlapping, components of Shannon information about the perceived stimulus. If these components do not overlap (as suggested by Stone, in preparation) then combining response time and accuracy data should provide better estimates of key parameters, which govern the decision process than relying on either response time or accuracy alone. However, our purpose here is not to make a theoretical innovation in decision modeling, but to use established decision models to demonstrate and quantify the benefits of decision modeling for experimentalists.

Previous authors have shown for specific paradigms and decisions that using decision models confers benefits beyond relying on speed, accuracy or some sub-optimal combination of the two, especially in the case of speed–accuracy trade-offs (Park and Starns, 2015; Zhang & Rowe, 2014). These results use data collected from participants in single experiments. Park and Starns (2015) show that for their data using decision models to estimate a drift parameter allows participant ability to be gauged separately from speed–accuracy trade-offs, and that these estimates consequently have higher predictive value. Zhang and Rowe (2014) used decision modeling to show that, for their data, it was possible to dissociate behavioral changes due to learning from those due to speed–accuracy trade-offs (revealing the distinct mechanisms of these two processes). In contrast to these studies, our approach is to use simulated data of multiple experiments so as to interrogate the value of decision models across a wide range of possibilities.

Ravenzwaaij, Donkin, and Vandekerckhov (2017, henceforth vRDV) have considerable sympathy with the approach we adopt here. They show that the EZ model, for across variations in participant number, trial number and effect size, has higher sensitivity to group differences than the full diffusion model, which they ascribe to its relative simplicity (a striking illustration of the bias/variance trade-off in model fitting, Yarkoni & Westfall, 2017).

### Contribution of the current work

Our work extends prior work in a number of ways. Our fundamental comparison is in the sensitivity of model parameters compared to behaviorally observed measures (RT, accuracy). Our purpose is not to compare different ‘measurement models’ (Ravenzwaaij et al., 2017), but to illustrate the benefits for experimentalists of using any decision model over analyzing a singular behavioral measure (reaction time or accuracy in isolation). We use the EZ model, for reasons of computational efficiency, and because prior work has shown that in most circumstances it preserves the benefits of fuller decision modeling approaches. We also confirm that the basic pattern of results holds for other model fitting methods, the HDDM (Wiecki, Sofer, & Frank, 2013) and fast-dm (Voss & Voss, 2007). We simulate null group effects and so can show false alarm rates as well as calculate results in terms of d’. Our use of d’ allows quantitative comparison and estimation of size of benefit across different speed–accuracy conditions. We explore the *combined* effects of group shifts in both drift and boundary, and so can show implications of speed–accuracy trade-offs between groups, alongside drift differences. As with all modeling work, the results we present have always been latent in existing models. Our focus is not on theoretical innovation, but in drawing out the implications of established models in a way that reveals the extent of their value and so promotes their uptake. For a discussion of the contribution of elaborating the consequences of existing models see Stafford (2009, 2010).

Our results are translated into the power-sample size space, which is familiar to experimental psychologists. Our results are accompanied by an interactive data explorer to aid in the translation of the value of decision models into a form most easily comprehendible by experimentalists. For these reasons, we hope that the current work can make a contribution in allowing experimentalists with less model-fitting experience to readily apprehend the large benefits of model fitting for decision-making data.

## Method

**Fig. 2:**
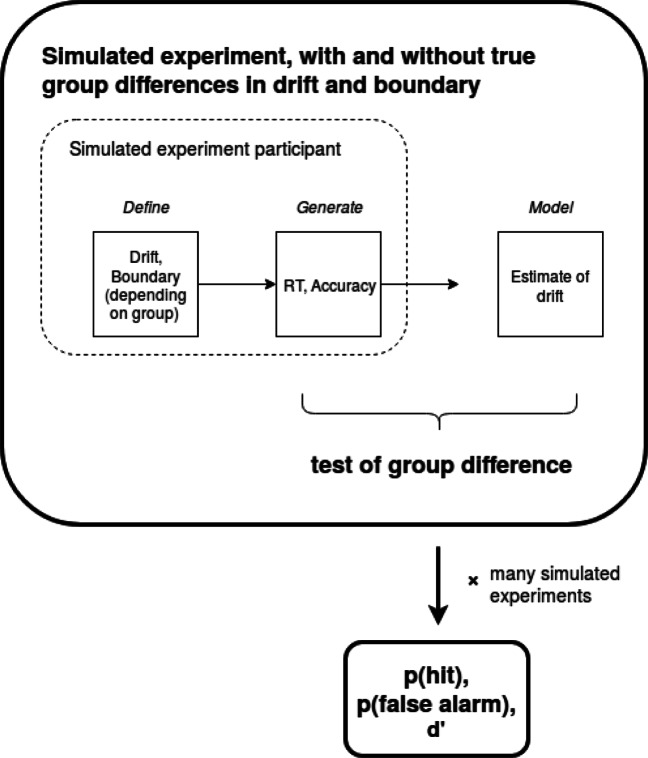
Overview of method: A between-groups experiment is simulated whereby there simulated participants have decision parameters (drift and boundary separation) sampled from defined distributions. From these defined distributions, which contain either a true or null difference between groups, simulated participants are sampled. From these sampled participant-level parameters, simulated decisions are generated, using the DDM, which generates behavioral outcome variables (RT and accuracy). For each participant, these outcome variables are modeled using the DDM to recover an estimated drift parameter. A test of group differences is then performed on the generated accuracy and RTs and on the estimated drifts. This is compared to the known difference in drift to categorize the test as correctly detecting a true difference between groups in participant discrimination (a hit), or incorrectly detecting a difference when there is none (a false alarm). Over many simulated experiments, and a range of parameter values for simulated sample size and size of true group differences in drift, the average probability of a hit and a false alarm, and the sensitivity (d’) are calculated

The broad approach is to consider a simple standard experimental design: a between-groups comparison, where each group contains a number of participants who complete a number of decision trials, providing both response time and accuracy data. We simulate data for true and null differences in drift rate between the groups, as well as true and null differences in boundary between the groups. By varying the number of simulated participants, we generate a fixed number of ‘scenarios’ defined by true/null effects in ability (drift) between groups, true/null SATOs (boundary) between groups and experiment sample size. We keep the number of decision trials per participant constant for all these analyses. For each scenario, we simulate many virtual experiments and inspect the behavioral measures to see how sensitive and specific they are to true group differences. We also fit the DDM and estimate the participant drift parameters, similarly asking how sensitive and specific estimates of drift are to true group differences. An overview of the method is illustrated in Fig. 2.

### Decision modeling

To generate simulated response data, we use the hierarchical drift diffusion model (HDDM, 2013). The toolbox can also perform model fitting, which uses Bayesian estimation methods to simultaneously fit individual decision parameters and the group distributions from which they are drawn.

While the HDDM offers a principled and comprehensive model fitting approach, it is computationally expensive. An alternative model fitting method, the EZ-DDM (Wagenmakers, Van Der Maas, & Grasman, 2007) offers a simple approximation, fitting a decision model with a smaller number of parameters, assuming no bias towards either of the two options and no inter-trial variability. This allows an analytic solution which is computationally cheap. Furthermore, the EZ-DDM has been shown to match the full DDM for a range of situations (Ravenzwaaij et al., 2017).

For the model fitting presented here (Figs. 5–8), we use the EZ-DDM, although initial exploration using both the HDDM and the fast-dm (Voss & Voss, 2007, a third model fitting framework) found qualitatively similar results, so our current belief is that these results do not depend on the particular decision model deployed from the broad class of accumulator models.^2^

Obviously, where we wish to simulate many thousands of independent experiments there are significant speed gains from parallelization. Parallelization was done by Mike Croucher, and the code run on University of Sheffield High Performance Computing cluster. A sense of the value of parallelization can be had by noting the data shown in, for example, Fig. 8 would have taken around one calendar month to generate on a single high-performance machine, even though they use the computationally ‘cheap’ EZ-DDM method. Python code for running the simulations, as well as the output data, figures and manuscript preparation files, is here 10.5281/zenodo.2648995.

### Analysis

Because we are not generating a comprehensive analytic solution for the full DDM we cannot claim that our findings are true for all situations. Our aim is merely to show that, for some reasonable choices of DDM parameters, using decision modeling is a superior approach to analyzing response time or accuracy alone, and to quantify the gain in statistical power.

To be able to make this claim of relevance of our simulations to typical psychology experiments, we need to be able to justify that our parameter choice is plausible for a typical psychology experiment. In order to establish this, we pick parameters which generate response times of the order of 1 s and accuracy of the order 90%. Each participant contributes 40 trials (decisions) to each experiment. Parameters for drift and boundary separation are defined for the group and individual participant values for these parameters are drawn from the group parameters with some level of variability (and, in the case of true effects, a mean difference between the group values, see below for details).

**Fig. 3:**
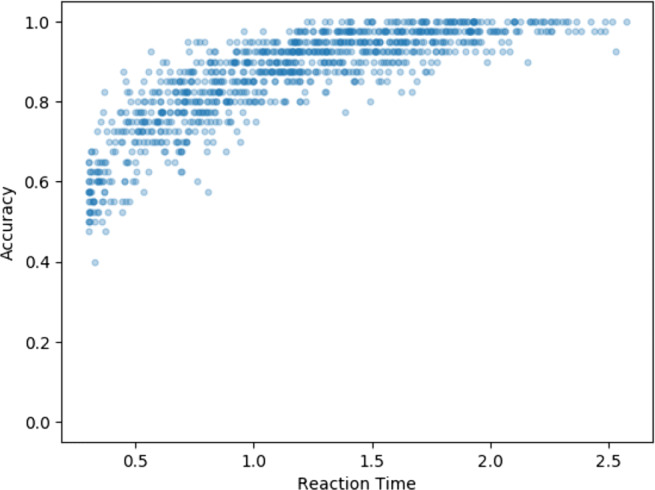
Directly visualizing the speed–accuracy trade-off: average response time and accuracy from a single simulated participant with all decision parameters kept fixed except for boundary separation, which is drawn from a normal distribution (mean = 2, variance = 1). 1000 simulated experiments, each of 40 trials

To illustrate this, we show in Fig. 3 a direct visualization of the speed–accuracy trade-off, by taking the base parameters we use in our simulated experiments and generating a single participant’s average response time and accuracy, using 1000 different boundary separation values. This shows the effect of varying boundary separation alone, while all other decision parameters are stable.

#### Simulating experimental data

For each scenario, we simulate a large number of experiments, testing a group (“A”) of participants against another group (“B”), with each participant contributing 40 trials. Participant parameters (most importantly the drift rate and boundary parameters) are sampled each time from distributions defined for each of the two simulated experimental groups, A and B. For the simulations with no true difference in sensitivity between A and B the drift rate of each group has a mean of 2 and within-group standard deviation of 0.05. For the simulations with a true difference in drift group B has a mean of 2 + *δ*, where *δ* defines an increase in the mean drift rate; the within-group standard deviations remain the same. For the simulations where there is no SATO, the mean boundary parameter is 2, with a within-group standard deviation of 0.05. For the simulations where there is a SATO, the boundary parameter of group B has an average of 2 − *δ*, where *δ* defines the size of the decrease in the mean boundary; the within-group standard deviations remain the same.

All simulations assume a non-decision time of 0.3 s, no initial starting bias towards either decision threshold and the inter-trial variability parameters for starting point, drift and non-decision time set to 0. Sample sizes between 10 and 400 participants were tested, moving in steps of ten participants for samples sizes below 150 and steps of 50 for samples sizes above 150. For each sample size 10,000 simulated experiments were run (each of 40 simulated participants in each of two groups).

#### Effect sizes, observed and declared

The difference between two groups can be expressed in terms of Cohen’s *d* effect size—the mean difference between the groups standardized by the within-group standard deviation. For the observed variables, response time and accuracy, effect sizes can only be observed since these arise from the interaction of the DDM parameters and the DDM model which generates responses. For drift rate, the difference between groups is declared (by how we define the group means, see above). The declared group difference in drift rate produces the observed effect size in response time and accuracy (which differ from each other), depending on both the level of noise in each simulated experiment, and the experiment design, particularly on the number of trials per participant. Experiment designs which have a higher number of trials per participant effectively sample the true drift rate more accurately, and so have effect sizes for response time and accuracy which are closer to the “true”, declared, effect size in drift rate.

**Fig. 4:**
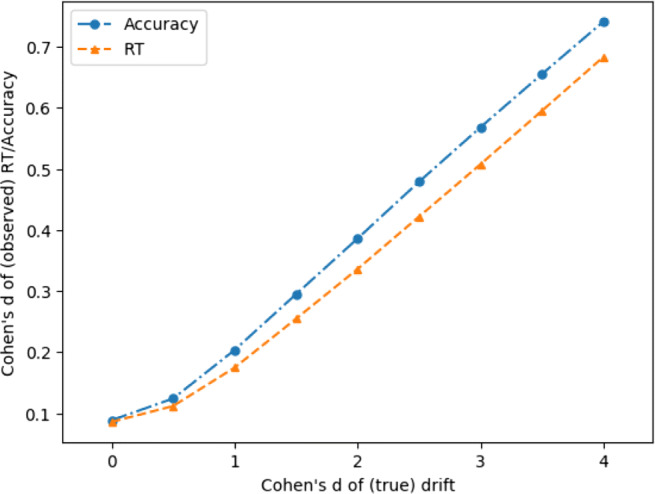
How differences in drift convert to observed differences in response time and accuracy (40 trials per ppt). Effect sizes for observed behavioral measures that are within the range typically observed by experimentalists are generated by larger actual differences in the underlying decision parameters

This issue sheds light on why decision modeling is more effective than analyzing response time or accuracy alone (because it recovers the generating parameter, drift, which is more sensitive to group differences), and why there are differences in power between measuring response time and accuracy (because these variables show different observed effect sizes when generated by the same true different in drift rates). Figure 4 shows how declared differences in drift translate into observed effect sizes for response time and accuracy.

#### Hits (power) and false alarms (alpha)

For each simulated experiment, any difference between groups is gauged with a standard two-sample *t* test.^3^ Statistical power is the probability of your measure reporting a group difference when there is a true group difference, analogous to the “hit rate” in a signal detection paradigm. Conventional power analysis assumes a standard false positive (*alpha*) rate of 0.05. For our simulations, we can measure the actual false-alarm rate, rather than assume it remains at the intended 0.05 rate.

For situations where only the drift differs between two groups, we would not expect any significant variations in false-alarm rate. However, when considering speed–accuracy trade-off changes between groups (with or without drift rate differences as well) the situation is different. This means that it is possible to get false positives in tests of a difference in drifts between groups because of SATOs. Most obviously, if a SATO means one group prioritizes speed over accuracy, analysis of response time alone will mimic an enhanced drift rate, but analysis of accuracy alone will mimic degraded drift rate. Ideally, the DDM will be immune to any distortion of estimates of drift rates, but that is what we have set out to demonstrate so we should not assume.

The consequence of this is that it makes sense to calculate the overall sensitivity, accounting for both the false-alarm rate, as well as the hit rate. A principled way for combining false alarm and hit rate into a single metric is d’ (“d prime”), which gives an overall sensitivity of the test, much as we would calculate the sensitivity independent of bias for an observer in a psychophysics experiment (Green & Swets, 1966).

## Results

The results shown here support our central claim that decision modeling can have substantial benefits. To explore the interaction of power, sample size, effect size, and measure sensitivity, we have prepared an interactive data explorer which can be found here https://sheffield-university.shinyapps.io/decision_power/ (Krystalli & Stafford, 2019, May).

### Without speed–accuracy trade-offs

For an idea of the main implications, it is sufficient to plot a slice of the data when the true difference in drift is a Cohen’s *d* of 2. Recall from Fig. 4 above that although this is a large difference in terms of the generating parameter, drift, this translates into small observed effect sizes in accuracy and response time (approximately 0.3–0.4, reflecting ‘medium’ effect sizes).

**Fig. 5:**
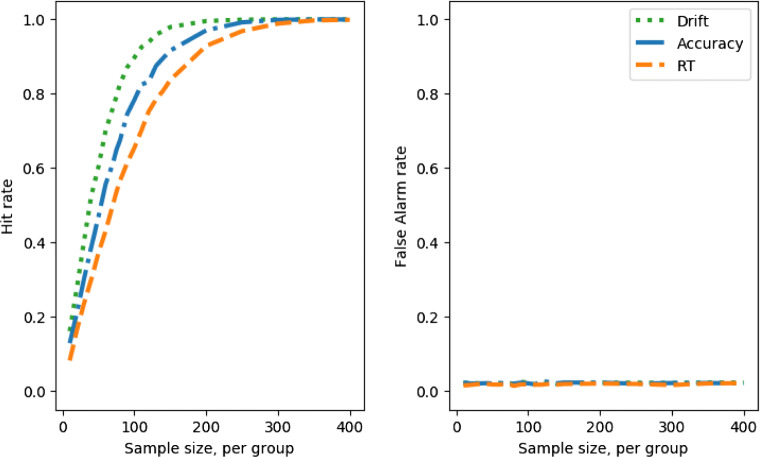
Hit rate and false-alarm rate against simulated experiment sample size, no speed–accuracy trade-off; comparing a between groups Cohen’s *d* effect size in the drift parameter of 2 (*left*) with 0 (*right*)

Figure 5, left, shows how sample size and hit rate interact for the different measures. The results will be depressingly familiar to any experimentalist who has taken power analysis seriously—a sample size far larger than that conventionally recruited is required to reach adequate power levels for small/medium group differences.

From this figure, we can read off the number of participants per group required to reach the conventional 80% power level (equivalent to hit rate of 0.8, if we assume a constant false-positive rate). For this part of the parameter space, for this size of difference between groups in drift, and no speed–accuracy trade-off, $\sim $140 participants are required to achieve 80% power if the difference between groups is tested on the speed of correct responses only. If the difference between groups is tested on the accuracy rate only, then $\sim $115 participants per group are required. If speed and accuracy are combined using decision modeling, and difference between groups is tested on the recovered drift parameters, then we estimate that $\sim $55 participants per group are required for 80% power. An experimentalist who might have otherwise had to recruit 280 (or 230) participants could therefore save herself (and her participants) significant trouble, effort, and cost by deploying decision modeling, recruiting half that sample size and still enjoying an increase in statistical power to detect group differences.

Figure 5, right, shows the false-alarm rate. When the difference in drifts is a Cohen’s *d* of 0, i.e., no true difference, the *t* tests on response time and accuracy both generate false-alarm rates at around the standard alpha level of 0.05.

**Fig. 6:**
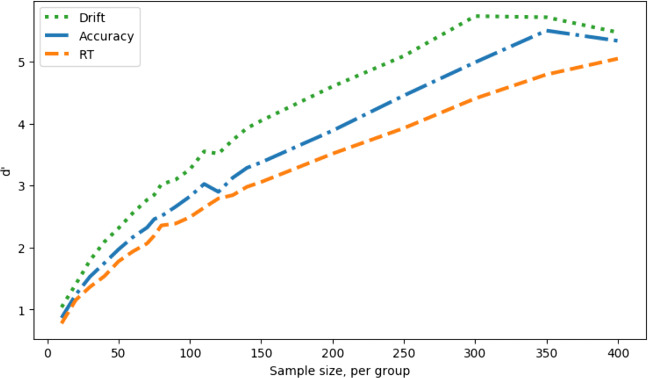
Measure sensitivity (d’) against simulated experiment sample size, no speed–accuracy trade-off; comparing a between-groups Cohen’s *d* effect size in the drift parameter of 2 (hits) with 0 (false alarms)

Figure 6 shows the measure sensitivity, d’ for each sample size. In effect, this reflects the hit rate (Fig. 5, left) corrected for fluctuations in false-alarm rate (Fig. 5, right). This correction will be more important when there are systematic variations in false-positive rate due to SATOs. Note that the exact value of d’ is sensitive to small fluctuations in the proportions of hits and false alarms observed in the simulations, and hence the d’ curves are visibly kinked despite being derived from the apparently smooth hit and false-alarm curves.

### With SATOs

The superiority of parameter recovery via a decision model becomes even more stark if there are systematic speed–accuracy trade-offs. To see this, we re-run the simulations above, but with a shift in the boundary parameter between group A and group B, such that individuals from group B have a lower boundary, and so tend to make faster but less accurate decisions compared to group A. On top of this difference, we simulate different sizes of superiority of drift rate of group B over group A.

For the plots below, the drift rate difference is, as above in the non-SATO case, 0.1 (which, given the inter-individual variability translates into an effect size of 2). The boundary parameter difference is also 0.1, a between group effect size 2.

**Fig. 7:**
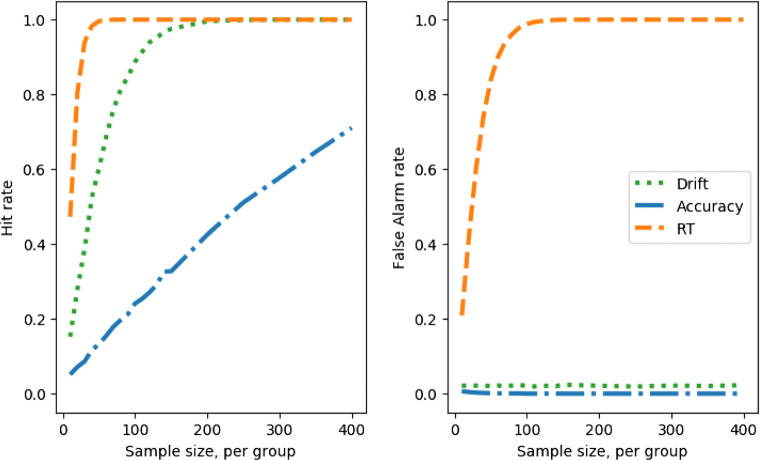
Hit rate and false-alarm rate against simulated experiment sample size, with a speed–accuracy trade-off (lowered decision boundary); comparing a between groups Cohen’s *d* effect size in the drift parameter of 2 (*left*) with 0 (*right*), and the boundary parameter also differing between groups with a Cohen’s *d* effect size of 2

Unlike the case where there are no SATOs, the response time measure is now superior for detecting a group difference over the drift measure; Fig. 7, left.

This, however, is an artifact of the SATO. If the boundary shift had been in the reverse direction then accuracy, not response time, would appear the superior measure (see below). Once we compare the false-positive rate, the danger of using a single observed measure becomes clear, Fig. 7, right.

When using the drift parameter as a measure, the SATO between the groups does not induce false alarms. The accuracy measure is insensitive so also does not suffer (but would if the boundary shift was in the opposite direction). The response time measure is catastrophically sensitive to false alarms, approaching 100% false-alarm rate with larger samples.

**Fig. 8:**
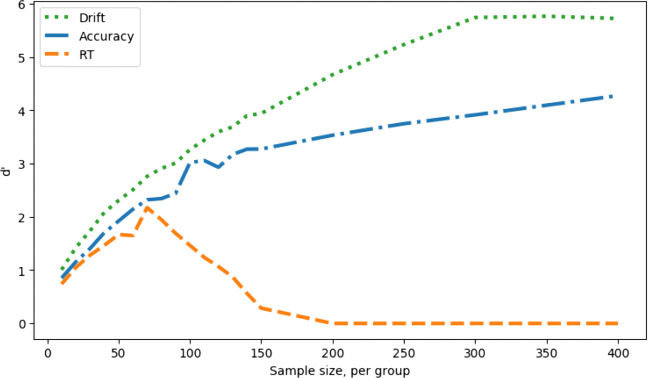
Measure sensitivity (d’) against simulated experiment sample size, no speed–accuracy trade-off (lowered decision boundary); comparing a between groups Cohen’s *d* effect size in the drift parameter of 2 (hits) with 0 (false alarms) as well as a between-groups Cohen’s *d* effect size of 2 in the boundary parameter

Figure 8 shows d’, which combines hit rate and the false-alarm rate, shows that the best measure overall is drift rate, as it is in the no-SATO case.

**Fig. 9:**
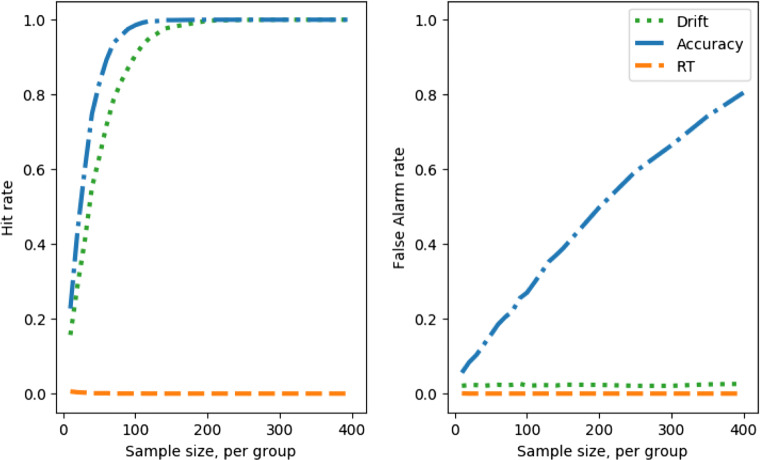
Hit rate and false-alarm rate against simulated experiment sample size, with a speed–accuracy trade-off (raised decision boundary); comparing a between- groups Cohen’s *d* effect size in the drift parameter of 2 (*left*) with 0 (*right*), and the boundary parameter also differing between groups with a Cohen’s *d* effect size of 2

**Fig. 10:**
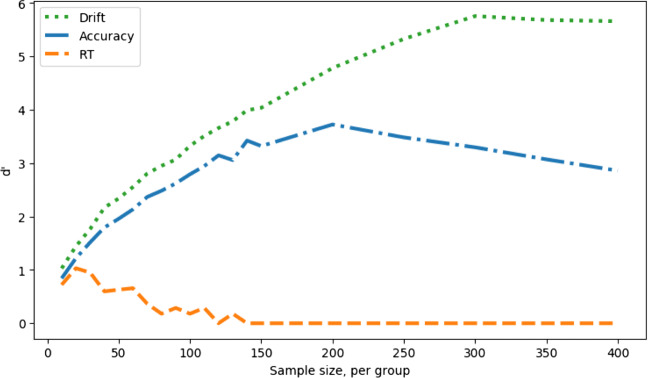
Measure sensitivity (d’) against simulated experiment sample size, no speed–accuracy trade-off (raised decision boundary); comparing a between-groups Cohen’s *d* effect size in the drift parameter of 2 (hits) with 0 (false alarms) as well as a between-groups Cohen’s *d* effect size of 2 in the boundary parameter

To confirm our intuitions concerning the effect of a raised decision boundary, as a opposed to a lowered one, we repeat the simulations with the boundary raised up by the same amount as it was lowered for the results shown in Figs. 7 & 8. The results are shown in Figs. 9 & 10. Comparing Fig. 9 with Fig. 7, we can see that, with a raised boundary, the accuracy appears the superior measure if hits alone are considered (left), but not if false alarms are taken into account (right). With the boundary raised, and hence more conservative responses, response time is less sensitive to group differences. As with the lowered boundary, it is possible to combine hits and false alarms in a single d’ measure (Fig. 10), which shows the same superiority of the estimated drift measure in comparison to both raw behavioral measures.

## Discussion

### Main conclusions

We have shown the benefits of fitting response time and accuracy data with standard decision models. Such decision models allow the estimation of the generating parameters of simple perceptual decisions, such that the participants’ sensitivity and response conservativeness are deconfounded. This allows more powerful tests of between-group differences, given a set sample size and/or the reduction in required sample for a set statistical power. Some insight into why decision modeling brings these benefits can be gained from Fig. 3. Here we show that the speed–accuracy trade-off exists as the decision threshold is shifted, and that it has a non-linear shape. Combining speed and accuracy not only provides more information, but cannot be done directly, but instead is best done via an accurate model of the underlying decision processes (such as the DDM).

*Inter alia* our results show that accuracy can be a more sensitive measure than response time given decision parameters which reasonably reflect a typical experiment. This confirms, in simulation, the result of Ratcliff and McKoon (2008) whose analysis of 18 experimental data sets showed that accuracy better correlated with participant drift rate than response time. Our results also provide some insight into why this is. Figure 4 shows that standard between-group effect size is more closely matched by generated accuracy than generated response times.

In the presence of systematic shifts in the speed–accuracy trade-off, this approach offers protection against false positives or false negatives (in the case that SATOs disguise true differences in sensitivity). Interestingly, under the parameter range used in these simulations, calculation of the d’ sensitivity measure shows that accuracy outperforms response time for SATO in both directions (whether more liberal, Fig. 8, or more conservative, Fig. 10).

We do not claim to make theoretical innovation in decision modeling—the work deploys widely used decision models ‘off the shelf’ and seeks to quantify the extent of the benefit for experimentalists of deploying decision modeling on their behavioral data. The extent of the statistical power gain is considerable. The exact benefit will vary according to the phenomenon and populations investigated, as well as experimental design. For the example design and parameter regime we showcase here, the use of decision modeling allows total sample size to be halved while still increasing statistical power. To explore the relation of sample size and effect size to the sensitivity of behavioral measures, and the decision modeling measures, we provide an interactive data explorer here https://sheffield-university.shinyapps.io/decision_power/ (Krystalli & Stafford, 2019, May).

### Qualifications

The results we showcase here and in the data explorer hold only for the parameter regime chosen. We have not analytically proved that parameter recovery with the DDM will always provide a statistical power gain. We have chosen a simple experimental design, with a plausible trial numbers per participant and decision parameters which generate realistic values for speed and accuracy of responses, but it is possible that for smaller effects, at the boundaries of maximum or minimum speed or accuracy, and/or with higher within and between participant noise, that decision modeling may not have the benefits depicted here (although it may also have greater benefits than those depicted here as well).

We have chosen not to explore a within-participants design because the issue of systematically different speed–accuracy trade-offs between conditions seems, *prima facie*, less likely. For between-groups designs, we know of several prominent cases where systematic SATOs confounded conclusions. For example, Pirrone, Dickinson, Gomez, Stafford, and Milne (2017) found that an apparent impairment of perceptual judgment among autism spectrum disorder (ASD) participants could be attributed to a difference in their SATO. The ASD group responded more conservatively, but decision modeling showed they had equivalent sensitivity to the non-ASD group. Ratcliff, Thapar, and McKoon (2006) found an analogous result for young vs. old participants on perceptual discrimination and recognition memory tasks.

We expect the statistical power gains of decision modeling to apply to within-participants designs. All other things being equal, between-groups designs have lower statistical power than within-participants designs, so it is for between-groups designs, which we assume an experimentalist would only deploy if they had no alternative, that decision modeling brings the greatest gains.

As well as occupying a particular point in the parameter space of decision models, our results are also generated using a particular model and model-fitting approach (the EZ-DDM, Wagenmakers et al., 2007), although we have verified that the same qualitative pattern can be produced by alternative approaches (Voss & Voss, 2007; Wiecki et al., 2013). Additionally, it is worth noting that for some parameterizations several prominent decision models are equivalent (Bogacz et al., 2006). A recent collaborative analysis project found that despite a large diversity of fitting methods, common inferences were made across different decision models (Dutilh et al., 2016). A reasonable conclusion from this project was that in many circumstances, the simple models should be preferred (Lerche & Voss, 2016). Ratcliff and Childers (2015) claim that hierarchical Bayesian methods of fitting, as used by the HDDM are best, at least for individual difference investigations (although see Jones & Dzhafarov, 2014 who claim that many variants of the DDM cannot be successfully distinguished by empirical measurement). Although we have not verified this, we expect to obtain similar results with many established models of decision-making, e.g., the LBA (Brown & Heathcote, 2008) or the LCA (Usher & McClelland, 2001), since we have no reason to suspect that our results are only dependent on the specific decision-making model used and rather depend on the established ability of a wide class of decision models to capture the regularities in behavioral data from human decisions.

### Wider context

As well as power gains, and protection against SATO confounds, decision modeling has other benefits to offer the experimentalist. It allows differences between participants or groups to be localized to particular components of the decision process. Decision modeling, since it relies on the distribution of responses rather than just the means, can also reveal underlying differences when single variables (e.g., response time) are stable (White, Ratcliff, Vasey, & McKoon, 2010).

There is a growing awareness of the limitations of studying only speed or accuracy alone (Oppenheim, 2017). A recent meta-analysis confirms a low correlation between speed and accuracy costs in psychological tasks (Hedge, Powell, Bompas, Vivian-Griffiths, & Sumner, in press). Vandierendonck (2017) compares seven transformations which combine reaction time and accuracy, without use of decision modeling, but finds none unambiguously superior either to the others or to inspecting raw reaction time and accuracy.

### Related work

A recent paper (Hedge, Powell, & Sumner, 2018) used a comparable simulation-based approach and reached a similar conclusion to ours—that model-free transformations of reaction time and accuracy, even if hallowed by common usage, are outperformed by a model-based transformation, which assumes a sequential sampling model like the DDM.

White, Servant, and Logan (2018) also present a parameter recovery account, but compare different variations of the sequential sampling models which are designed to account for decisions under conflict. Their focus is on comparing between different decision models rather than model-free and model-based transformations of reaction time and accuracy.

Baker et al., (2019) used the simulation method to address a question of equal importance to experimentalists—how does the number of trials interact with sample size to affect statistical power? Like us, they present an interactive demonstration of their findings https://shiny.york.ac.uk/powercontours/

### Getting started with decision modeling

Those who wish to apply decision models to their data have a range of tutorials and introductory reviews available (Forstmann, Ratcliff, & Wagenmakers, 2016; Voss, Nagler, & Lerche, 2013), as well as statistical computing packages which support model fitting (Voss & Voss, 2007; Wiecki-etal:2013). Although analyzing speed and accuracy data with decision models incurs a technical overhead, we hope we have made clear the considerable gains in both enhanced sensitivity to true differences and protection against spurious findings that it affords.

### Conclusions

Decision modeling offers large benefits to the experimentalist, and is based on a principled framework that has seen substantial validation and exploration. No analysis plan can rescue an ill-conceived study, and experimentalists have many other considerations which can enhance statistical power before they attempt decision modeling (Lazic, 2018). Our attempt here has just been to illustrate how, in cases where speed and accuracy are collected from two groups of participants, decision modeling offers considerable power gains, and the attendant increased chances of discovering a true effect and/or reduction of required sample size, without increased risk of false positives. The contribution this paper hopes to make concerns the size of these benefits. These are not just, as could be theoretically shown, non-zero, but they are, under conditions which it is realistic to expect to hold for a typical experiment, consequential and so worthy of the experimentalist’s consideration.

## Abbreviations

DDM -: Drift diffusion model
SATO -: Speed–accuracy trade-off

## Footnotes

1 Note that we choose to cite work by the lead author here for illustration, rather than highlight any other researchers for their use of these suboptimal practices.

2 Computational constraints mean that systematically confirming this by fully exploring the parameter space presented in this manuscript must be future work.

3 Note, for high-accuracy values, *t* tests may not be appropriate (they are strictly not applicable to proportions anyway, but this may become a real issue for values very close to 1 or 0).

## References

[CR1] Baker, D.H., Vilidaite, G., Lygo, F.A., Smith, A.K., Flack, T.R., Gouws, A.D., & Andrews, T.J. (2019). Power contours: Optimising sample size and precision in experimental psychology and human neuroscience. arXiv Preprint arXiv:1902.06122. 10.1037/met0000337 PMC8329985 32673043

[CR2] Bezeau S, Graves R (2001). Statistical power and effect sizes of clinical neuropsychology research. Journal of Clinical and Experimental Neuropsychology.

[CR3] Bogacz R, Brown E, Moehlis J, Holmes P, Cohen JD (2006). The physics of optimal decision making: A formal analysis of models of performance in two-alternative forced-choice tasks. Psychological Review.

[CR4] Brown SD, Heathcote A (2008). The simplest complete model of choice response time: Linear ballistic accumulation. Cognitive Psychology.

[CR5] Bruyer R, Brysbaert M (2011). Combining speed and accuracy in cognitive psychology: Is the inverse efficiency score (IES) a better dependent variable than the mean reaction time (RT) and the percentage of errors (PE)?. Psychologica Belgica.

[CR6] Button KS, Ioannidis JP, Mokrysz C, Nosek BA, Flint J, Robinson ES, Munafò MR (2013). Power failure: Why small sample size undermines the reliability of neuroscience. Nature Reviews Neuroscience.

[CR7] Cohen J (1962). The statistical power of abnormal-social psychological research: a review. The Journal of Abnormal and Social Psychology.

[CR8] Davidson D, Martin AE (2013). Modeling accuracy as a function of response time with the generalized linear mixed effects model. Acta Psychologica.

[CR9] Dutilh, G., Annis, J., Brown, S.D., Cassey, P., Evans, N.J., Grasman, R.P., & et al. (2016). The quality of response time data inference: A blinded, collaborative assessment of the validity of cognitive models. *Psychonomic Bulletin & Review*, 1–19. 10.3758/s13423-017-1417-2 PMC6449220 29450793

[CR10] Fitts PM (1966). Cognitive aspects of information processing: III. Set for speed versus accuracy. Journal of Experimental Psychology.

[CR11] Forstmann BU, Ratcliff R, Wagenmakers E-J (2016). Sequential sampling models in cognitive neuroscience: Advantages, applications, and extensions. Annual Review of Psychology.

[CR12] Geuter, S., Qi, G., Welsh, R.C., Wager, T.D., & Lindquist, M.A. (2018). Effect size and power in fMRI group analysis. arXiv:295048.

[CR13] Gold JI, Shadlen MN (2001). Neural computations that underlie decisions about sensory stimuli. Trends in Cognitive Sciences.

[CR14] Gold JI, Shadlen MN (2002). Banburismus and the brain: Decoding the relationship between sensory stimuli, decisions, and reward. Neuron.

[CR15] Green, D.M., & Swets, J.A. (1966). *Signal detection theory and psychophysics*. Wiley.

[CR16] Hedge C, Powell G, Sumner P (2018). The mapping between transformed reaction time costs and models of processing in aging and cognition. Psychology and Aging.

[CR17] Hedge, C., Powell, G., Bompas, A., Vivian-Griffiths, S., & Sumner, P. (in press). Low and variable correlation between reaction time costs and accuracy costs explained by accumulation models: Meta-analysis and simulations. *Psychological Bulletin*. 10.1037/bul0000164 PMC6195302 30265012

[CR18] Heitz RP (2014). The speed–accuracy tradeoff: History, physiology, methodology, and behavior. Frontiers in Neuroscience.

[CR19] Ioannidis JP (2005). Why most published research findings are false. PLoS Medicine.

[CR20] Jones M, Dzhafarov EN (2014). Unfalsifiability and mutual translatability of major modeling schemes for choice reaction time. Psychological Review.

[CR21] Krystalli, A., & Stafford, T (2019, May). Interactive web application accompanying paper ’quantifying the benefits of using decision models with response time and accuracy data’. 10.15131/shef.data.8109161, https://figshare.shef.ac.uk/s/11f65856db28308644a4. 10.3758/s13428-020-01372-w PMC7575468 32232739

[CR22] Lazic SE (2018). Four simple ways to increase power without increasing the sample size. Laboratory Animals.

[CR23] Lerche V, Voss A (2016). Model complexity in diffusion modeling: Benefits of making the model more parsimonious. Frontiers in Psychology.

[CR24] Lerche V, Voss A (2017). Retest reliability of the parameters of the Ratcliff diffusion model. Psychological Research Psychologische Forschung.

[CR25] Liesefeld HR, Janczyk M (2019). Combining speed and accuracy to control for speed–accuracy trade-offs (?). Behavior Research Methods.

[CR26] Liesefeld HR, Fu X, Zimmer HD (2015). Fast and careless or careful and slow? Apparent holistic processing in mental rotation is explained by speed–accuracy trade-offs. Journal of Experimental Psychology: Learning, Memory, and Cognition.

[CR27] Lovakov, A., & Agadullina, E (2017, November). Empirically derived guidelines for interpreting effect size in social psychology. 10.17605/OSF.IO/2EPC4.

[CR28] Maxwell SE (2004). The persistence of underpowered studies in psychological research: Causes, consequences, and remedies. Psychological Methods.

[CR29] Open Science Collaboration (2015). Estimating the reproducibility of psychological science. Science.

[CR30] Oppenheim GM (2017). A blind spot in correct naming latency analyses. Cognitive Neuropsychology.

[CR31] Palmer J, Huk AC, Shadlen MN (2005). The effect of stimulus strength on the speed and accuracy of a perceptual decision. Journal of Vision.

[CR32] Park J, Starns JJ (2015). The approximate number system acuity redefined: A diffusion model approach. Frontiers in Psychology.

[CR33] Pashler H, Wagenmakers E (2012). Editors’ introduction to the special section on replicability in psychological science: A crisis of confidence?. Perspectives on Psychological Science.

[CR34] Pirrone A, Stafford T, Marshall JA (2014). When natural selection should optimize speed–accuracy trade-offs. Frontiers in Neuroscience.

[CR35] Pirrone A, Dickinson A, Gomez R, Stafford T, Milne E (2017). Understanding perceptual judgment in autism spectrum disorder using the drift diffusion model. Neuropsychology.

[CR36] Pirrone A, Azab H, Hayden BY, Stafford T, Marshall JA (2018). Evidence for the speed–value trade-off: Human and monkey decision making is magnitude sensitive. Decision.

[CR37] Ratcliff R (1978). A theory of memory retrieval. Psychological Review.

[CR38] Ratcliff R, Childers R (2015). Individual differences and fitting methods for the two-choice diffusion model of decision making. Decision.

[CR39] Ratcliff R, McKoon G (2008). The diffusion decision model: Theory and data for two-choice decision tasks. Neural Computation.

[CR40] Ratcliff R, Rouder JN (1998). Modeling response times for two-choice decisions. Psychological Science.

[CR41] Ratcliff R, Thapar A, McKoon G (2006). Aging and individual differences in rapid two-choice decisions. Psychonomic Bulletin & Review.

[CR42] Ratcliff R, Smith PL, McKoon G (2015). Modeling regularities in response time and accuracy data with the diffusion model. Current Directions in Psychological Science.

[CR43] Ratcliff R, Smith PL, Brown SD, McKoon G (2016). Diffusion decision model: Current issues and history. Trends in Cognitive Sciences.

[CR44] Ravenzwaaij Dvan, Donkin C, Vandekerckhove J (2017). The EZ diffusion model provides a powerful test of simple empirical effects. Psychonomic Bulletin & Review.

[CR45] Sedlmeier P, Gigerenzer G (1989). Do studies of statistical power have an effect on the power of studies?. Psychological Bulletin.

[CR46] Seli P, Jonker TR, Cheyne JA, Smilek D (2013). Enhancing SART validity by statistically controlling speed–accuracy trade-offs. Frontiers in Psychology.

[CR47] Silberzahn, R., Uhlmann, E.L., Martin, D., Anselmi, P., Aust, F., Awtrey, E.C., & et al. (2017). Many analysts, one dataset: Making transparent how variations in analytical choices affect results.

[CR48] Simmons JP, Nelson LD, Simonsohn U (2011). False-positive psychology: Undisclosed flexibility in data collection and analysis allows presenting anything as significant. Psychological Science.

[CR49] Smith PL, Ratcliff R (2004). Psychology and neurobiology of simple decisions. Trends in Neurosciences.

[CR50] Stafford, T (2009). What use are computational models of cognitive processes? In *Connectionist models of behaviour and cognition II*: World Scientific.

[CR51] Stafford, T (2010). How do we use computational models of cognitive processes? In *Connectionist models of neurocognition and emergent behavior: From theory to applications* (pp. 326–342): World Scientific.

[CR52] Stafford T, Ingram L, Gurney KN (2011). Piéron’s law holds during Stroop conflict: Insights into the architecture of decision making. Cognitive Science.

[CR53] Stanley, T., Carter, E.C., & Doucouliagos, H (2017). What meta-analyses reveal about the replicability of psychological research. Working paper, Deakin Laboratory for the Meta-Analysis of Research. Retrieved from https://www.deakin.edu.au/_data/assets/pdf_file/0007/1198456/WhatMeta-AnalysesReveal_WP.pdf.

[CR54] Stone JV (2014). Using reaction times and binary responses to estimate psychophysical performance: an information theoretic analysis. Frontiers in Neuroscience.

[CR55] Szucs D, Ioannidis JP (2017). Empirical assessment of published effect sizes and power in the recent cognitive neuroscience and psychology literature. PLoS Biology.

[CR56] Teodorescu AR, Moran R, Usher M (2016). Absolutely relative or relatively absolute: Violations of value invariance in human decision making. Psychonomic Bulletin & Review.

[CR57] Townsend, J.T., & Ashby, F.G. (1983). Stochastic modeling of elementary psychological processes. CUP Archive.

[CR58] Usher M, McClelland JL (2001). The time course of perceptual choice: The leaky, competing accumulator model. Psychological Review.

[CR59] Vandierendonck A (2017). A comparison of methods to combine speed and accuracy measures of performance: A rejoinder on the binning procedure. Behavior Research Methods.

[CR60] Voss A, Voss J (2007). Fast-dm: A free program for efficient diffusion model analysis. Behavior Research Methods.

[CR61] Voss A, Nagler M, Lerche V (2013). Diffusion models in experimental psychology: A practical introduction. Experimental Psychology.

[CR62] Wagenmakers E-J, Van Der Maas HL, Grasman RP (2007). An ez-diffusion model for response time and accuracy. Psychonomic Bulletin & Review.

[CR63] White CN, Ratcliff R, Vasey MW, McKoon G (2010). Using diffusion models to understand clinical disorders. Journal of Mathematical Psychology.

[CR64] White CN, Servant M, Logan GD (2018). Testing the validity of conflict drift-diffusion models for use in estimating cognitive processes: A parameter-recovery study. Psychonomic Bulletin & Review.

[CR65] Wickelgren WA (1977). Speed–accuracy tradeoff and information processing dynamics. Acta Psychologica.

[CR66] Wiecki TV, Sofer I, Frank MJ (2013). HDDM: Hierarchical Bayesian estimation of the drift-diffusion model in python. Frontiers in Neuroinformatics.

[CR67] Yarkoni T, Westfall J (2017). Choosing prediction over explanation in psychology: Lessons from machine learning. Perspectives on Psychological Science.

[CR68] Yates, D., & Stafford, T (2018, June). ’Cognitive strategy’ in visual search: How it works and when it generalises. 10.17605/OSF.IO/5DUP8.

[CR69] Zhang J, Rowe JB (2014). Dissociable mechanisms of speed–accuracy tradeoff during visual perceptual learning are revealed by a hierarchical drift-diffusion model. Frontiers in Neuroscience.

